# Rapid Tests for Viral Upper Airway Respiratory Infections in the Workplace: A Pilot Study on a Professional Football Team

**DOI:** 10.3390/medicina61061072

**Published:** 2025-06-11

**Authors:** Dimitrios Papagiannis, George D. Vavougios, Kyriakos Yiangou, Evangelos Latzourakis, Foteini Malli, Konstantinos I. Gourgoulianis, Georgios M. Hadjigeorgiou

**Affiliations:** 1 Public Health and Adults Immunization Laboratory, Department of Nursing, University of Thessaly, 41500 Larissa, Greece; 2 Department of Neurology, Medical School, University of Cyprus, Nicosia 2029, Cyprus; vavougyios.georgios@ucy.ac.cy (G.D.V.); hadjigeorgiou.georgios@ucy.ac.cy (G.M.H.); 3 Private Health Sector Nicosia Cyprus, Nicosia 2042, Cyprus; yiangouk@hotmail.com; 4 Department of Health Sciences, University of Nicosia, Nicosia 2417, Cyprus; latzourakis.e@unic.ac.cy; 5 Department of Nursing, University of Thessaly, 41500 Larissa, Greece; mallifoteini@yahoo.gr; 6 Department of Respiratory Medicine, Faculty of Medicine, University of Thessaly, 41500 Larissa, Greece; kgourg@uth.gr

**Keywords:** football players, flu, RSV, adenovirus, respiratory infections, absenteeism

## Abstract

*Background and Objectives:* Acute infections among elite athletes are predominantly attributed to upper respiratory tract pathogens. From a practical standpoint, medical personnel responsible for the healthcare of professional football players should be aware of this and develop infection prevention strategies. This pilot study aimed to investigate the prevalence of respiratory infections in football players using multiplex rapid diagnostic tests targeting four respiratory pathogens. *Materials and Methods:* The mean age of the participants was 32.76 ± 10.96 years. Among the participants, 32 were professional football players, with a mean age of 26.5 years, SD + 5.3, and 18 were members of staff, with a mean age of 44.3 years, SD + 8.6. In the present study, participants were followed up over a period of 6 months (from October 2024 to March 2025). *Results:* Among the participants and among a total of 1078 tests, 10 tests were found to be positive. We recorded a proportion of 0.46% for Flu-A, 0.27% for Flu-B, 0.18% for SARS-CoV-2, and 0 positive tests for RSV and adenovirus. There were six days of absence for players and staff and the proportion of total absenteeism was calculated as 3.7%. Univariate analysis revealed no statistically significant difference in infection risk between staff and players (odds ratio: 0.3795; 95% confidence interval: 0.07843–1.735). *Conclusions:* The multiplex rapid diagnostic test platform has a demonstrated ease of use and appears to be a reliable and safe method for distinguishing contagious symptomatic individuals from non-contagious individuals in occupational settings. Early identification of respiratory infections facilitates improved clinical management, thereby enhancing the quality of care for both athletes and supporting staff.

## 1. Introduction

Upper respiratory tract infections (URTIs) are among the most prevalent acute illnesses affecting elite athletes, particularly those engaged in high-intensity sports such as professional football. The effective prevention and management of these infections are critical responsibilities for medical teams overseeing the health of professional athletes. These infections primarily involve the nasal passages, throat, and sinuses and are typically caused by a variety of viruses, including rhinoviruses, coronaviruses, and influenza viruses. Due to the high physical and psychological stress experienced by athletes, their immune systems may be temporarily compromised, increasing susceptibility to URTIs. Additionally, factors such as close contact in team sports, travel, and environmental exposures further contribute to the risk of infection. Understanding the epidemiology, pathophysiology, and preventive measures of viral URTIs in athletes is essential to optimize health management and maintain peak athletic performance. Research in exercise immunology indicates that intense and prolonged physical exertion may transiently suppress immune function, thereby increasing susceptibility to respiratory infections. This immunosuppression is characterized by alterations such as neutrophilia, lymphopenia, and decreased natural killer cell activity [1,2,3]. Timely diagnosis and rapid assessment of infection severity, ideally without reliance on time-consuming clinical tests, are essential for preventing the spread and progression of URTIs among athletes. Although various pathogens—including viruses, bacteria, fungi, and mycoplasma—can cause respiratory infections, viruses are the predominant etiological agents. Human rhinovirus (HRV) is most commonly associated with URTIs, accounting for approximately 10–40% of cases. Other significant viral pathogens include influenza viruses, respiratory syncytial virus (RSV), and coronaviruses [4].

Numerous studies have investigated the incidence of URTIs across different athletic disciplines, including endurance sports (e.g., running, cycling, cross-country skiing, swimming, rowing) and team sports (e.g., rugby, wheelchair basketball) [5,6,7,8,9,10]. Athletes typically present with a range of symptoms, such as nasal congestion, rhinorrhea, sore throat, and swollen lymph nodes. Lower respiratory tract involvement may manifest as cough, wheezing, and chest pain. In some cases, systemic symptoms like fever, general fatigue, and myalgia are observed. Infections can occur during various phases of training and competition, including pre-competition, competition, and recovery periods [11,12].

The prompt diagnosis of infections and early assessment of disease severity, without reliance on time-intensive clinical testing, is critical for mitigating both transmission and disease progression. While a variety of pathogens—including bacteria, fungi, and mycoplasmas—can induce infections, viruses are responsible for the majority of respiratory tract infections. Among these, human rhinovirus (HRV) is the most frequently implicated, accounting for approximately 40% of cases. Other commonly detected respiratory viruses include influenza viruses, respiratory syncytial virus (RSV), and coronaviruses, which follow HRV in prevalence [13].

Meanwhile, in the recent pandemic, the athletes followed the general restrictions that were announced by the governments for lockdown and social distance. Studies that analyzed the incidence of COVID-19 infections recorded a low incidence rate during a one-year period of observation, with the majority of cases being asymptomatic. Systematic weekly monitoring and readily available laboratory evaluation of samples with Real-Time Polymerase Chain Reaction (RT-PCR) kept the incidence low for the staff and players in comparison to the general population [14,15].

The use of rapid antigen detection tests (RADTs) in occupational settings can complement, but not replace, occupational health and safety measures and existing non-pharmaceutical interventions at the workplace aimed at preventing the introduction and spread of respiratory diseases. Diagnosing these infections promptly is crucial for better clinical outcomes but can be challenging. Rapid antigen tests for multiple pathogens offer a reliable, fast, and cost-effective method for detecting viral infections. Early and accurate diagnosis can help reduce the misuse of antibiotics and enable the use of antiviral treatments, as well as mitigate the absence from work and the resulting loss of productivity [16]. In high-contact and performance-critical environments like workplaces and sports settings, multiple rapid tests performed regularly and in combination offer an efficient, scalable, and cost-effective method for early detection and transmission prevention. This strategy enhances safety, preserves productivity, and maintains competitive integrity in sports. During the recent pandemic, rapid antigen detection tests significantly contributed to the overall COVID-19 testing capacity, offering advantages in terms of shorter turnaround times and reduced costs, especially in situations in which RT-PCR testing capacity was limited. In April 2021, the European Agency for Safety and Health at Work (EU-OSHA) conducted a survey among its national Focal Points (FOPs) to gather information on the use of rapid antigen tests in a workplace context [17]. Common criteria mentioned by the European Centre for Disease Prevention and Control (ECDC) for the choice of RADTs used included test sensitivity ≥90% and CE certification [18].

The absenteeism rate, also referred to as the absence rate or absence percentage, represents the proportion of unscheduled absences attributable to illness or other non-preplanned causes. This metric can be calculated at the individual, team, or organizational level, serving as an important indicator of organizational health and workforce stability.

To the best of our knowledge, this study is the first to examine risk reduction in high-contact environments like a sport team for four viral pathogens via rapid tests. Workplaces, especially where physical distancing is limited, are at higher risk of outbreaks. In sports, the risks are magnified by close physical interaction, shared facilities, and frequent travel. Regular rapid testing minimizes downtime, protects athletes and staff, and prevents event cancelations. Little is known about the impact of annual viral respiratory infection among professional footballers. In the present cross-sectional study, we aimed to explore viral infections among football players via rapid tests for four respiratory pathogens in a pilot study on a professional football team in Cyprus. This is the first study conducted in Cyprus on professional athletes and football team staff. All participants provided appropriate weekly nasal swabs to test for the detection of viral respiratory pathogens. Furthermore, the absenteeism rate is a key measure of organizational health and well-being. In the present study, we tried to calculate the absenteeism rate for an organization like a professional football team.

## 2. Materials and Methods

Our study reports on a viral upper airways respiratory infection monitoring program lasting for six months conducted among 32 professional football players and 18 staff personnel of an elite professional football team in Cyprus. The study period was between 1 October 2024 and 31 March 2025. According to the schedule, every week before the Friday training, the medical staff collected nasopharyngeal samples from the players and staff. Participants were informed about the aims of this study and that data from the research protocol would be treated anonymously and would be assigned a special code number by the medical staff. Medical staff were contacted by the authors before the start of the championships to participate in this study. They were provided with instructions on collecting data and completing weekly forms correctly using Excel Microsoft 2019. The study was conducted according to the guidelines of the Declaration of Helsinki and approved firstly by the Nursing Department 52/2-2-2024 and secondly by the Institutional Review Board (or Ethics Committee) of the Nursing Department of the University of Thessaly (approved with protocol number 42/21-12-2024). Informed consent was obtained from all participants. Collected specimens were subjected to antigen testing for the identification of four distinct pathogens. To enhance procedural efficiency, a simplified sample collection protocol was implemented, utilizing a single swab to detect all four pathogens rather than requiring separate samples for each. This streamlined method reduces patient discomfort, lowers associated costs, decreases personnel workload, and minimizes procedural stress for both patients and healthcare staff. Qualitative variables were summarized using absolute and relative (%) frequencies, while quantitative variables were expressed as means with corresponding standard deviations. The chi-square test (χ^2^) was employed for univariate analysis of categorical variables. Odds ratios (ORs) and their corresponding 95% confidence intervals (CIs) were calculated to assess associations. A *p*-value of <0.05 was considered indicative of statistical significance. All statistical analyses were conducted using Microsoft Excel 2019, (MSO, 16.0.10417.20012), (Microsoft Corporation, Redmond, WA, USA) and Open Epi (version 3.01).

### Principles Underlying the Antigen Testing Method

Antibodies specific to viral proteins are applied to the nitrocellulose membrane’s test line region. During testing, the antibodies affixed to gold nanoparticles interact with the viral antigens present in the samples. As the substance moves upward through the test membrane and joins the membrane’s immobilized antibodies, a colored line forms in the test region. The presence of this vibrant line signifies a favorable result. A colorful line in the control zone serves as a procedural control that consistently indicates whether the test was finished correctly. The results of this study’s tests were interpreted after fifteen minutes. The patient’s head is tilted back 70 degrees. A sterile swab is removed from the pouch. While being gently rotated, the swab is inserted less than one inch (about 2 cm) into the patient’s nostril (until resistance is met at the turbinates). The swab is rotated five times against the nasal wall and is then slowly removed from the nostril. Using the same swab, the collection procedure is repeated with the second nostril. After the specimen collection, the swab is placed in the extraction tube by rotating the swab forcefully against the side of the tube for 1 min. The best results are obtained when the specimen is vigorously extracted in the solution. The swab is removed, squeezing the sides of the tube to extract as much liquid as possible. The swab is discarded. The extraction tube is closed with the dropper cup. Two drops are added in the circular window of the cassette for each test. The four rapid diagnostic tests (RDTs) used in this study were manufactured by PROGNOSIS BIOTECH S.A (Larissa, Greece) and met the requirements of EN ISO 13485:2016 [19].

These tests were used in a previous cross-sectional study published recently and demonstrated high sensitivity and specificity [20]. The sensitivity of the FLU A and B test was 91.15% (95% CI: 84.33–95.67%), and the specificity was 98.96% (95% CI: 97.86–99.58%). The sensitivity and specificity of the adenovirus and RSV test was {92.45% (95% CI: 81.79–97.91%), 99.32% (95% CI: 98.41–99.78%)} and {92.59% (95% CI: 75.71–99.09%), 99.47% (95% CI: 98.65–99.86%)}, respectively. Lastly, the sensitivity of the SARS-CoV-2 test was 100.00% (95% CI: 79.41–100.00%) and the specificity was 99.74% (95% CI: 99.06–99.97%) [20]. The present pilot study aimed to record the proportion of influenza A/B, SARS-CoV-2, RSV, and adenovirus cases using rapid antigen tests among professional football players and staff during winter 2025 in Cyprus.

The absenteeism rate is a key measure of organizational health and well-being. Whether it is measured for the entire organization or an individual, knowing the exact absence rate will help shape interventions. In the current study, we tried to calculate the absence rate according to ISO norms [21]. We used the following Absenteeism Rate Formula to calculate the total absenteeism of athletes and staff:

$$\frac{Total\;number\;of\;absences/days}{Total\;period\;of\;time/days}\times 100=X\%$$

The outcome variable was the number of absences, and we included the number of potential workforce days as an offset variable to express the outcome as a rate. As covariates, we included the time elapsed (to capture the trend over the period).

## 3. Results

The collected specimens included 50 adults, consisting of 49 men and 1 woman. The mean age of the participants was 32.76 ± 10.96 years. Among the participants, 32 were professional football players, with a mean age of 26.5 years, SD ± 5.3, and 18 were members of staff, with a mean age of 44.3 years, SD ± 8.6. In the present pilot study, participants were under surveillance over a period of 6 months (from October 2024 to March 2025). Among the participants and among a total of 1078 tests, 10 tests were positive. We recorded a proportion of 0.46% (N = 5) for Flu-A, 0.27% (N = 3) for Flu-B, 0.18% (N = 2) for SARS-CoV-2, and 0 positive tests for RSV and adenovirus (Table 1). Out of the 10 cases that turned out to be positive, 9 cases were symptomatic (90%) and 1 (10%) was asymptomatic. Symptoms were recorded as mild and often included fever, head and body aches, coughing, and a stuffy or runny nose. Other than minimal symptomatic care, none of the athletes or personnel who tested positive or reactive during the research period needed hospitalization or further medical attention, with the exception of medical advice provided on-site by medical staff for an absence of 1 or 2 days, depending on the severity of symptoms (Table 1).

There were six days of absence among the players and staff, and the proportion of total absenteeism was calculated to be 3.7%. The first cases of absence recorded, in the 49th week of the year, were the first confirmed case of Flu-A, with symptoms, and one case of SARS-CoV-2, without symptoms. The second absences recorded were in the 3rd week, with two confirmed cases of Flu-A with symptoms: one player and one member of staff. In both cases, 2 days’ absence from training was suggested by the medical team. Finally, in the 5th week, we recorded two cases of Flu-B with symptoms, with the same suggestion of two days’ absence (Table 2).

We recorded eight cases of Flu-A and B, with a total point prevalence of 16%, during the observation period. The first cases were recorded in (W 49), with two cases of Flu-A, and the last case of Flu-B was recorded in the 5th week of 2025. The majority of the recorded cases were symptomatic. In addition, we recorded two cases of SARS-CoV-2 in the 49th and 51st weeks of observation.

On univariate analysis of flu infection among players and staff, no significant differences were recorded between staff and football players in terms of getting ill (odds ratio: 0.3795) (95% CI: (0.07843–1.735)).

## 4. Discussion

In this pilot study, we evaluated the feasibility and effectiveness of a low-cost, rapid diagnostic surveillance system for monitoring viral respiratory infections in a professional football team in Cyprus. Our primary aim was to assess the utility of point-of-care testing for four common viral respiratory pathogens over a six-month winter period, with a focus on infection rates and the associated absenteeism among players and staff. Monitoring and preventing infections is a critical component of athlete health management, particularly in team sports, where close contact increases transmission risk. This study aligns with international public health recommendations, which highlight the need for resilient, population-based surveillance systems for respiratory viral pathogens such as influenza viruses, SARS-CoV-2, RSV, and emerging threats.

Although the existing literature suggests that athletes may experience infections more frequently than the general population [22], the evidence remains inconclusive [14,23]. One possible explanation for this uncertainty may be methodological. Athletes are more likely to seek medical attention for minor infections than non-athletes, and they may monitor their health more closely due to the potential impact that even mild illnesses can have on training and performance.

Our results support this concept, as we recorded a low proportion of respiratory infections during the winter of 2024–2025 among football players and staff in comparison to the general population; furthermore, increased awareness and direct access to medical staff enhanced the protective wall for players and staff, respectively. We recorded proportions of 0.46% for Flu-A and 0.27% for Flu-B. According to the ECDC, at the EU/EEA level, an influenza primary care test positivity threshold of 10% was reached at the start of the influenza season. In a recent report published on 2 April 2025, comprising an overview of respiratory virus epidemiology in the European Union/European Economic Area EU/EEA, week 13 of the winter season in the EU/EEA was characterized by an intense influenza season, and overall influenza activity peaked in week 6; decreasing trends are now being observed in each of the influenza A(H1), A(H3), and B/Vic viruses. Most countries have moved from an early season dominated by influenza A to A/B co-dominance or B dominance, while for a small number, the opposite now applies. Decreasing trends in influenza activity are being observed in almost all countries, with half reporting having returned to baseline or low levels of intensity. Influenza A(H3) and B viruses were most commonly reported in week 13 [24]. Our results fully agree with ECDC data on the epidemiology of the flu in Europe: our first cases were recorded in the 49th week of the year, with two cases of Flu-A, and the last case of Flu-B was recorded in the 5th week of 2025.

Our results differ from ECDC data for RSV. According to European data, in the last winter, a concurrent respiratory syncytial virus (RSV) epidemic was observed [24]. During a six-month period, no case of RSV was recorded via rapid test among the subjects who participated in the present study. The most reasonable explanation for this substantial difference is the age allocation, as the highest prevalence in the general population is usually observed among infants and older adults. We recorded a very low proportion for SARS-CoV-2, with two cases and a total positivity rate of (2/1078, 0.18%). Both cases were recorded among members of staff, and the participants had mild symptoms. Our results agree with the ECDC data for SARS-CoV-2, where activity remained at low levels in all countries for the same observation period [24].

In general, adenovirus infections are self-limiting and mild and often require no specific treatment beyond symptomatic management [25]. Infections among athletes are relatively rare. However, a notable case report described a previously healthy collegiate football player who developed acute liver failure and rhabdomyolysis in the context of an adenovirus infection, marking the first documented instance of adenovirus-associated liver failure in a young, healthy adult [26].

In the current study, no adenovirus cases were recorded during the observation period. Nonetheless, adenovirus-related outbreaks have been documented in athletic populations. For instance, a study from Korea reported an outbreak of acute respiratory infection due to adenovirus in the swimming department of a physical education school [27]. Similarly, an earlier outbreak of pharyngoconjunctivitis among swimming athletes occurred during a competitive event in the Peloponnese region of southern Greece [28].

Absenteeism, particularly among athletes, is typically defined as absence from training or competition due to illness or injury [29]. In a competitive environment such as professional football, there is a potential cost to the club team derived from that situation (illnesses and/or injuries, psychological momentum, etc.), and the athlete may lose their position on the team. Our results support a low absenteeism rate for athletes during the winter of 2024–2025 due to viral respiratory infections, and all cases of absence followed suggestions from medical staff to prevent the possibility of spreading the disease to players and staff.

Importantly, 90% of the recorded cases in our study were symptomatic. This contrasts with findings from previous PCR-based studies, where a majority of positive cases were asymptomatic. This highlights the role of symptom-based testing strategies in resource-limited settings. While molecular tests remain the gold standard, our findings support the practicality and value of rapid antigen testing in sports environments lacking advanced diagnostic infrastructure [14]. Since it enables the prompt identification and distinction of primary respiratory infections, the simultaneous detection of numerous pathogens utilizing particular quick tests is essential. In workplaces where molecular testing may not be available, rapid antigen tests provide a useful substitute for prompt diagnosis. In the present study, the confirmation of illness was based on a combination of clinical symptoms and RADTs’ results.

Multiple rapid testing is not just a health intervention but a strategic asset in managing the safety, continuity, and resilience of workplaces and sports ecosystems. It enables a proactive response, protects human capital, and sustains organizational operations in the face of infectious threats. Although many infection control measures are well-established and widely recognized, adherence to these protocols is critical to the overall effectiveness of mitigation strategies. Historically, compliance has been monitored and enforced by football federations; however, this responsibility should now be distributed among athletes, medical and support personnel, team officials, and technical staff. The most significant advancement to be emphasized is that the success of future infection prevention efforts is fundamentally dependent on the consistent commitment and compliance of all participants and attendees in implementing these countermeasures. In conclusion, our study demonstrates that implementing a rapid, low-cost surveillance system using point-of-care tests can effectively monitor respiratory infections in elite athlete populations. This approach not only supports athlete health and performance but also aligns with broader public health goals for the early detection and containment of respiratory viral pathogens. To enhance the present results, in future, further investigations need to include all professional football teams in Cyprus with expanded parameters like vaccination coverage for vaccine-preventable diseases and illness detection with abbreviated symptom questionnaires; biochemical assessments should be performed by the team physicians or by medical staff.

## 5. Conclusions

In the present study, we present the proportion of four types of viral respiratory infections in a professional football team population observed in the winter period of 2024–2025. We report a low rate of respiratory infections and absenteeism in professional football players during the last winter. Our findings show that the use of multiple RADTs’ could be a valuable and safe diagnostic method for viral respiratory diseases. Multiple RADTs’ were also demonstrated to be an important triage tool for medical staff in professional football teams and during competitions. Overall, using multiple RADTs’ is an effective strategy for reducing the spread of respiratory diseases in the workplace and contributes to outbreak control. Multiple RADTs’ are an easy-to-use diagnostic platform and are developing into a safe approach for distinguishing symptomatic contagious individuals with respiratory diseases from non-contagious individuals at the workplace. The present study has several limitations. First, the study’s cross-sectional design makes it impossible to draw conclusions about the causation of the relationships that were found, and the results may be considered insufficiently informative for drawing such conclusions. The small number of participants is not representative of all football players and staff in Cyprus. Despite the high quality of the RADTs’ used in the present study, as confirmed by our previous study [20], the lack of confirmation by Polymerase Chain Reaction is a limitation of our results.

## Figures and Tables

**Table 1:** Characteristics of the participants and cases of viral infections.

	N, %, Number of Tests		Total Positivity Rate
Male	49	98.0%	
Female	1	2.0%	
Football players	32	64%	
Age, mean	26.5 SD 5.3		
Staff	18	36%	
Age, mean	44.3 SD 8.6		
Flu-A	5/1078		0.46
Flu-B	3/1078		0.27
SARS-CoV-2	2/1078		0.18
RSV	0		-
Adenovirus	0		-
Cases with symptoms	9/10	90%	

**Table 2:** Days of absenteeism among players and staff per month in the six-month observation period.

Period 2024–2025	Football Players	Staff	Days of Absence
October	0	0	0
November	0	0	0
December	1	1	2
January	1	0	2
February	0	2	2
March	0	0	0

## Data Availability

The data presented in this study are available on request from the corresponding author due to reasonable request.

## Abbreviations

The following abbreviations are used in this manuscript:

URTIs	Upper Respiratory Tract Infections
HRV	Human Rhinovirus
RSV	Respiratory Syncytial Virus
PCR	Polymerase Chain Reaction
RADTs	Rapid Antigen Detection Tests
EU-OSHA	Agency for Safety and Health at Work
ECDC	European Centre for Disease Prevention and Control
EU/EEA	European Union/European Economic Area
